# Use of new technology to improve utilization and adherence to immunotherapy

**DOI:** 10.1186/1939-4551-7-29

**Published:** 2014-11-21

**Authors:** Smita Joshi, Ves Dimov

**Affiliations:** Department of Medicine, Weill Cornell Medical College, New York, NY 10065 USA; Department of Pediatrics, University of Chicago, Chicago, IL 60637 USA; Department of Medicine, University of Chicago, Chicago, IL 60637 USA

**Keywords:** Adherence, Immunotherapy, Technology, Internet, Social media

## Abstract

Technology and social media have dramatically altered the landscape in which we practice medicine. Clinicians have increasingly turned to technology and the internet to enhance patient care. Allergists have used these modalities to improve utilization and adherence to immunotherapy. Electronic medical records (EMRs) are being widely adopted by allergy practices and some offer allergy/immunology specific modules that aid in daily workflow. The development of specialized devices that reduce pain associated with immunotherapy administration may improve compliance with immunotherapy. Social media and other forms of electronic communication such as e-mail, Facebook, Twitter, short message service (SMS), and YouTube give clinicians multiple avenues to disseminate information and reach their patients, possibly improving patient adherence to therapy. Finally, tablet computers, online networks, and electronic surveys provide additional ways to connect patients and physicians.

## Introduction

A common challenge in the treatment of chronic illnesses is patient nonadherence to medication regimens. Studies on sublingual immunotherapy (SLIT) have demonstrated that 55% to 82% of patients are noncompliant with the recommended course of treatment [1]. Adherence to subcutaneous immunotherapy has varied widely ranging from 13%-89% [2]. Technological innovations and social media have significantly influenced dissemination of information across all areas of medicine. The use of these tools provides a potentially powerful avenue to educate patients and improve utilization and adherence to immunotherapy. The advantages and drawbacks of these tools are summarized in Table 1. Most of the technology discussed in the article is fairly new and therefore studies of each individual tool and its impact on adherence are limited or still in early stages.

**Table 1:** **Advantages and limitations of technology in immunotherapy**

Technology	Advantages	Limitations
Electronic Medical Records (EMRs)	-Improve efficiency and workflow	-No studies have evaluated if this modality improves utilization and adherence of immunotherapy
	-Allergy/immunology specific modules allow for management of injections, documentation of skin test results, and immunotherapy documentation and billing.	-Cost
	-Some modules can generate reports of patients late for injections and can create batch reminder e-mails/letters	
Commercially available devices aimed at reducing pain with injections	-Use of vibration in the vicinity of intended injection site supposedly reduces pain associated with injections	-No studies have evaluated if this modality improves utilization and adherence of immunotherapy
	-May improve patient satisfaction and adherence to treatment.	-Cost
Social Media		
Twitter	-Increasing presence of allergists using Twitter to disseminate information	-No studies have evaluated if these modalities improves utilization and adherence of immunotherapy
	-Twitter is used by allergists at the meetings of AAAAI, ACAAI, EAACI, and WAO	-Difficulty validating authors’ credentials and qualifications
YouTube	-Has been used by AAAAI, ACAAI, and EAACI to disseminate information	-Information anarchy
	-Allergists can use YouTube to post educational videos and then embed them into their website. They can also embed videos created by reputed health organizations	-Difficulty finding time to use social media by physicians
Online Networks	-Provide forum for allergists and patients to engage in real-time collaboration	-Dearth of active participation of physicians
		-Decreased physician workplace acceptance and support
		-Concerns over maintaining confidentiality
Short Message Service (SMS)/Text Messaging	-Daily SMS reminders have improved adherence of medication treatment outcomes in allergic rhinitis.	-No studies have evaluated if this modality improves utilization and adherence of immunotherapy
Electronic Surveys	-Have been used to assess immunotherapy practices and utilization	-No studies have evaluated if this modality improves utilization and adherence of immunotherapy
	-Have been used to assess characteristics of patients living with allergies	
Tablet Computers	-Graphic display and ease of interface	-No studies have evaluated if this modality improves utilization and adherence of immunotherapy
	-Point-of-care tablet computers have been shown to improve allergy/immunology education	

## Review

### Electronic Medical Records (EMRs)

EMRs are increasingly prevalent in medical practices due to their ability to improve efficiency and workflow. Some EMRs commonly used by allergists in the United States (US) include GE Centricity, Allscripts, EPIC, Meditab-IMS, NextGen, ModuleMD, eClinical Works, Rosch, and a free web-based EMR called Practice Fusion [3]. Many EMRs have allergy/immunology specific modules. Such software is tailored towards allergists’ needs and allows for features such as management of injections, documentation of skin test results, and immunotherapy documentation and billing. Some of these modules also include features that may improve immunotherapy adherence by generating reports of patients late for injections and creating batch reminder e-mails/letters. EMRs with both full versions as well as allergy/immunology and immunotherapy specific modules include Meditab/AllergyEHR [4], Module MD [5], and Mountainside Software [6]. Rosch Visionary Systems offers an immunotherapy module that can interface with an existing EMR [7]. Both the American Academy of Allergy, Asthma, and Immunology (AAAAI) and American College of Allergy, Asthma and Immunology (ACAAI) have online buyer’s guides that can help prospective clients find suitable EMRs [8]. Scientific societies provide guidance to their members regarding best practices for use of EMRs and other new technologies for improving adherence.

### New devices aim to decrease pain of subcutaneous immunotherapy

There are several commercially available devices aimed at reducing pain associated with injections and other invasive medical procedures. These devices claim to improve patient satisfaction and adherence to treatment regimens by “calming your nerves” about shots, making the experience “ouch-less”. In real life, experiences are variable. SofStic is a disposable single-use device based on the dental product Accupal [9]. It uses vibrations in the immediate vicinity of the intended injection to reduce pain by supposedly creating a small area of numbness that includes the injection site. The reusable wand is listed at $125 US dollars and a package of 100 disposable single use tips costs $200 US dollars. The Vibration Anesthesia Device by Blain Labs is another product that uses vibration to produce a local anesthetic effect, reportedly reducing pain associated with injections [10]. There are no PubMed listed studies of any device aimed to relieve pain from immunotherapy injections. Assessing whether these devices improve utilization and/or adherence to immunotherapy is an exciting direction for future research.

### Social media and other forms of electronic communications

Use of social media such as email, Facebook, Twitter, short message service (SMS) also known as text messaging, and YouTube continues to grow. Social media is a significant part of daily life for both adolescents and adults and allows for virtual relationships with peers sharing similar interests. Many allergists have welcomed social media into their practices. Social media has been adopted by allergy clinics in a variety of ways such as the use of Twitter to broadcast daily pollen counts and “allergy shot” hours, Facebook to post photos of the reception area and staff members, and YouTube videos to welcome and educate patients and the general public.

Electronic media offer a novel way to potentially improve allergy care. A survey sent to asthma patients between the ages of 12-40 revealed that e-mail was the most preferred method to receive asthma information and to communicate with a physician [11]. However, only 22% of participants expressed interest in either receiving information or communicating with a physician through Facebook [11]. Many of these individuals were concerned about using Facebook and other social media because of privacy and noted that their use of these sites were for social reasons rather than health information.

A 2012 survey of over 3000 American adults revealed that 85% own a cell phone, 80% of cell phone owners send or receive text messages, and 9% of cell phone users receive text updates or alerts about health or medical issues [12]. Medical professionals are increasingly finding a role for digital technologies such as text messaging in patient care.

The utility of text messaging was investigated in a randomized controlled trial of 50 patients with allergic rhinitis who were assigned to receive either a daily SMS reminder on their cell phone to take intranasal corticosteroid treatment or receive nothing [13]. This study found that self-reported adherence to medication in the SMS group was significantly higher than in the control group. This study suggests that daily SMS reminder improves adherence to medication and treatment outcomes in allergic rhinitis, however no data exist regarding SMS messaging in immunotherapy utilization and adherence. Additionally, this trial was only 30 days long. The effect of daily SMS on medication adherence is often lost after additional months, which would be detrimental for treatments that require long-term compliance such as immunotherapy.

Current studies of technology use in adolescents with asthma and allergy do not provide consistent evidence of effectiveness. The positive attitude toward use of social media or mobile technology opens the possibility for future studies to further explore the potential benefits of such interventions [14].

In today’s world, medical information is widely available through both print and online media. However, the sources of information that patients rely on are often written by non-medical professionals or are based upon data that lacks credibility or evidence. Both print and online media may overemphasize, underemphasize, or misportray certain aspects of disease, leading to patient misconceptions. Examination of twenty years of newspaper articles from the New York Times, Los Angeles Times, and Washington Post revealed that there was relatively little coherence in whether asthma was portrayed as directly caused by air pollution or triggered by exposures [15]. Furthermore, outdoor sources of air pollution were covered more frequently. Studies of print media coverage of immunotherapy have not been reported. Allergists must have active online presence to balance the print/online media coverage that may be incomplete or misguided.

While the rise of social media has given physicians multiple avenues to disseminate information, social media can also have negative consequences related to lapses of professionalism and the blurring of the lines between professional and personal life online [16]. Caution must be exercised when posting in public forums so as to not display private patient information or unprofessional behavior. State medical boards and society organizations have developed specific guidelines on social media use by physicians.

A recent study used semi-structured interviews to investigate the perspectives and experiences of physicians adopting social media in healthcare [17]. Lack of trust was a major challenge addressed by study participants. Since anyone can post to social media regardless of educational and professional qualifications, there is no way to validate an author’s credentials. Another barrier identified was information anarchy. This refers to a state where finding relevant information is challenging due to the disorganized and chaotic nature of information created and disseminated. It can be difficult to navigate through websites where both social and professional conversations are taking place between anyone from a patient to a medical expert upon a backdrop of advertisements, pop-up offers, and marketing banners. Other challenges identified included difficulty finding time to use social media, a lack of workplace acceptance and support, lack of active participation, and concerns surrounding maintaining confidentiality.

Used appropriately, social media offers a powerful channel for physician and patient education with almost unlimited distribution. Many scientific societies are active users of social media services for dissemination of quality scientific evidence and patient education materials. As a practical example, the professional websites of one of the authors have had more than 10 million page views and 5 million visitors since 2005, with 5,000 page views every day. With every hit of the “publish” button of the sites, the message reaches approximately 30,000 people daily including 30,000 RSS and email subscribers, 15,000 Twitter followers, 2,500 daily visitors, and 3,500 Facebook fans.

### Tablet computers for patient education

Technological advancements such as the development of tablet computers offer a novel way to disseminate information and educate patients. The graphic display and ease of interface makes a tablet computer such as the iPad™ or Android-based tablet a powerful tool. A 2011 study evaluated the utility of a point-of-care tablet computer (iPad™) for allergy/immunology education. iPads™ preloaded with educational diagrams and clinical photographs were used to educate patients about allergic rhinitis and conjunctivitis and different treatment options, including immunotherapy [18]. Of the 20 patients surveyed, 100% liked the iPad™ to help explain their or their children’s condition and 100% would like the iPad™ to be used again to help explain medical information. Patients’ comments included “Showing the pictures helps”, “It gave me the visual representations”, “It was professional”, “Having the decision trees to take home was helpful”, “I like it because you don’t have to use a lot of paper”, and “If [the pictures/information was] shown on a computer it wouldn’t have been as convenient and it’s much better than a verbal description“. While this data suggest that tablet computers improve patient education, future research should assess whether the use of a tablet computers improves utilization and adherence to immunotherapy.

### Twitter use by allergists for immunotherapy education and adherence to therapy

Twitter is a fast growing social network that allows for microblogging and disseminating short pieces of information - 140 characters for an individual post, called a tweet. Twitter has been used by medical professionals to engage with patients, stay up to date with medical literature and interact with colleagues [19]. Some allergists share allergy/immunology news and this can be used as a form of personalized continuing medical education (CME) which takes 10 minutes or less several times per week. Allergy practices also use Twitter to share daily pollen counts, work hours for immunotherapy (“allergy shots”) clinic, and physician on call information.

An analysis of allergist and immunologist use of Twitter conducted for one year (from May 2011 to May 2012) showed that 85 self-identified allergists were on Twitter in 2012 compared to 18 identified in the prior 2011 study [20]. This represents a 470% increase (more than 4-fold) in Twitter use by allergists in one year. Most allergists were located in the USA (91%), used their professional/personal name (95%) and had a profile picture (84%). There were 66 allergy-related organizations identified on Twitter. Eighty percent of the allergists had more than 50 followers, 64% followed more than 50 users, 79% had more than 20 tweets, and 78% of the allergists followed at least one allergist.

Allergists also use Twitter during the annual meetings of AAAAI, ACAAI, European Academy of Allergy and Clinical Immunology (EAACI), and World Allergy Organization (WAO). Physicians, patients, and the general public follow the meeting using the name of the meeting preceded by a hashtag, for example, #AAAAI. The 2012 AAAAI meeting had 5,041 registered delegates and 25 allergists (0.49% of the attendees) used Twitter to publish 2,650 tweets [21]. Their tweets reached 250,000 people, nearly 50 times the number of people who attended the meeting. Of the tweets, 1,397 (52.7%) were facts and 7.2% (192) were facts with links to support the factual information. There were 366 (13.8%) replies, 274 (10.3%) status updates, 219 (8.2%) retweets, 112 (4.2%) opinions, 46 (1.7%) queries and 25 (0.9%) advertisements. Social media, and Twitter in particular, is an efficient way to disseminate medical information to medical professionals and the public. A small subset of 25 allergists expanded the educational reach of the 2012 AAAAI annual meeting to 250,000 individuals.

While twitter activity by allergists has rapidly increased, no literature exists on the efficacy of physician tweets in improving patient education and medication compliance. This presents an opportunity for future studies to further explore the utility of Twitter in making a meaningful impact on such factors.

### YouTube use by allergists for immunotherapy education

YouTube is another platform that has been used to spread patient education on a wide variety of topics in medicine. However, as with all forms of social media, the credibility and quality of information available on YouTube varies considerably. An analysis of chronic obstructive pulmonary disease (COPD) patient education videos on YouTube found that while YouTube has the potential to reach an inform patients, existing video content and quality varies significantly. The high-quality videos were uploaded predominantly by reputable health organizations and qualified medical professionals, not by individual users [22]. There is a need for more reliable and accurate patient education videos by physicians and other qualified medical professionals. At present, organizations such as AAAAI, ACAAI, and EAACI have YouTube videos on immunotherapy. Allergists can use YouTube to post educational videos and then embed them in their website or blog. For improved efficiency, allergists can also embed YouTube videos created by reputable health organizations such as AAAAI, ACAAI, EAACI and WAO.

### Online networks for allergists and patients and their role for immunotherapy research

Online networks provide a forum for allergists and patients to connect with one another. There are prominent web-based networks in Italy that link allergy centers and help share their clinical protocols and epidemiologic data [23]. For example, the Hospital Allergy Net of Piemonte, Italy, was established in 2003 and connects multiple hospitals of the region through a web platform. A national network called the Italian Pediatric Allergy Network connects several Italian pediatric allergy units and adopts a web platform for observational and intervention studies on pediatric asthma and allergies.

In the US, an Allergy/Immunology (A/I) Interest Group was created at University of Chicago on Google+. The Google document with scholarly activity has been shared among 12 participants. Seven members have updated the document a total of 38 times and listed 28 unique projects. Since August 2011, members have generated and published 5 peer reviewed articles, 10 national abstract presentations, and 2 book chapters. Five members have successfully matched in allergy/immunology fellowship, 2 are currently applying, and 2 have future plans to apply.

### Electronic surveys assess immunotherapy practices and utilization

Electronic online surveys have been used to assess utilization of immunotherapy and practice patterns of allergen immunotherapy administration. SLIT is a widely used form of specific immunotherapy in Europe. Due to the lack of Food and Drug Administration (FDA)-approved SLIT products in the U.S., it has not been as widely adopted in the US. However, an electronic survey sent to practicing allergists in the US in 2011 surprisingly showed that the rates of SLIT use have nearly doubled in the last 4 years, with 11.4% of US respondents (59 out of 519 respondents) reporting SLIT use [24]. It is anticipated that once the FDA- approved product is available, there will be widespread use of SLIT in the US.

Allergen immunotherapy practice patterns have also been identified via electronic survey. A 2010 electronic survey was sent to allergists who are members of the AAAAI to examine the ways allergists administer immunotherapy [25]. The survey found that most of the 1201 responders practice in an urban or suburban practice in the US and have been in practice for greater than 10 years. Those in practice for greater than 10 years were more likely to adjust the dose and frequency of immunotherapy in pollen season.

In a recent study, online surveys were used to assess the impact of severe allergies on the lives of young patients [26]. Patient participants were recruited from a membership database, allergy clinics, and social media. Another online survey administered to members, website visitors, and social media followers of the Kids with Food Allergy Foundation showed that differences in beliefs and opinions about oral immunotherapy (OIT) existed between OIT participants and nonparticipants [27]. This demonstrated that thorough and accurate information about OIT safety, efficacy, risks, and approval status was not universally conveyed. Use of electronic surveys in the future may help clinicians identify barriers to immunotherapy administration and delivery such as inconsistent patient education.

## Conclusion

We are in an era where technology and social media are omnipresent and influence the daily lives of patients and physicians. Allergists are using these mediums to attempt to improve patient care and compliance to treatment regimens. While we are seeing an increase in use of technology and electronic communication channels by allergists, there is a paucity of information in the literature on how these interventions affect patient outcomes. Future studies should be directed towards investigating how technology and social media improve the utilization and adherence of immunotherapy.
